# Cimp-Positive Status is More Representative in Multiple Colorectal Cancers than in Unique Primary Colorectal Cancers

**DOI:** 10.1038/s41598-019-47014-w

**Published:** 2019-07-19

**Authors:** Sandra Tapial, Susana Olmedillas-López, Daniel Rueda, María Arriba, Juan L. García, Alfredo Vivas, Jessica Pérez, Laura Pena-Couso, Rocío Olivera, Yolanda Rodríguez, Mariano García-Arranz, Damián García-Olmo, Rogelio González-Sarmiento, Miguel Urioste, Ajay Goel, José Perea

**Affiliations:** 1 Digestive Cancer Research Group, 12 de Octubre Research Institute, Madrid, Spain; 2 Hereditary Cancer Laboratory, 12 de Octubre University Hospital, Madrid, Spain; 3 New Therapies Laboratory, Foundation Health Research Institute-Fundación Jiménez Díaz University Hospital, Madrid, Spain; 4 Department of Biochemistry, Gregorio Marañón University Hospital, Madrid, Spain; 5 Biomedical Research Institute of Salamanca (IBSAL), University Hospital of Salamanca-USAL-CSIC, Salamanca, Spain; 6 Institute of Molecular and Cellular Biology of Cancer (IBMCC), University of Salamanca-CSIC, Salamanca, Spain; 7 Surgery Department, University Hospital 12 de Octubre, Madrid, Spain; 8 Familial Cancer Clinical Unit, Human Cancer Genetics Program, Spanish National Cancer Research Centre (CNIO), Madrid, Spain; 9 Pathology Department, University Hospital 12 de Octubre, Madrid, Spain; 10 Surgery Department, Fundación Jiménez Díaz University Hospital, Madrid, Spain; 11 Center for Biomedical Network Research on Rare Diseases (CIBERER). Institute of Health Carlos III, Madrid, Spain; 12 Beckman Research Institute at City of Hope Comprehensive Cancer Center 1218S, Fifth Avenue, Monrovia, CA 91016 USA

**Keywords:** Cancer genomics, Colorectal cancer

## Abstract

Colorectal cancer (CRC) with CpG island methylator phenotype (CIMP) is recognized as a subgroup of CRC that shows association with particular genetic defects and patient outcomes. We analyzed CIMP status of 229 individuals with CRC using an eight-marker panel (*CACNA1G*, *CDKN2A*, *CRABP1*, *IGF2*, *MLH1*, *NEUROG1*, *RUNX3* and *SOCS1*); CIMP-(+) tumors were defined as having ≥ 5 methylated markers. Patients were divided into individuals who developed a “unique” CRC, which were subclassified into early-onset CRC (EOCRC) and late-onset CRC (LOCRC), and patients with multiple primary CRCs subclassified into synchronous CRC (SCRC) and metachronous CRC (MCRC). We found 9 (15.2%) CIMP-(+) EOCRC patients related with the proximal colon (p = 0.008), and 19 (26.8%) CIMP-(+) LOCRC patients associated with tumor differentiation (p = 0.045), MSI status (p = 0.021) and *BRAF* mutation (p = 0.001). Thirty-five (64.8%) SCRC patients had at least one CIMP-(+) tumor and 20 (44.4%) MCRC patients presented their first tumor as CIMP-(+). Thirty-nine (72.2%) SCRC patients showed concordant CIMP status in their simultaneous tumors. The differences in CIMP-(+) frequency between groups may reflect the importance of taking into account several criteria for the development of multiple primary neoplasms. Additionally, the concordance between synchronous tumors suggests CIMP status is generally maintained in SCRC patients.

## Introduction

Colorectal cancer (CRC) is one of the most frequent malignancies representing the second cause of mortality related to cancer, moreover its incidence continues to rise gradually^1^. Growing evidence suggests that CRC is a heterogeneous disorder that can develop through different pathways involving distinct combinations of genetic and epigenetic alterations. Specific phenotypes are derived from these alterations which result in different prognosis and disease evolution^2,3^. Consequently, a better knowledge of the molecular events involved in the appearance and progression of CRC could provide new insight into therapeutic targets and markers for risk stratification^4^. Currently, three main pathways are widely accepted to be involved in the etiology of CRC: Chromosomal Instability (CIN), Microsatellite Instability (MSI) and CpG Island Methylator Phenotype (CIMP)^5–7^. Structural rearrangements as well as gains and losses of chromosome fragments are characteristic features of CIN tumors, possibly associated with higher mutation rates; the majority of sporadic cases are in this group^8^. MSI is associated with changes in short microsatellite repeats, caused by deficient mismatch repair (MMR) genes, and is related to hereditary non-polyposis colorectal cancer (HNPCC), also called Lynch Syndrome (LS), and some sporadic cases in the elderly population^9^. Lastly, tumor suppressor and DNA repair genes are frequently transcriptionally silenced in CIMP cases. Furthermore, several studies have shown that CIMP positivity is associated with proximal colon location, presence of mucinous features, poor tumor differentiation, MSI, female gender and high *BRAF* mutation rates^10,11^.

Genetic, biological and clinical differences have been identified depending on the age of onset of CRC in many studies, thus it has been suggested that CRC should be subclassified attending this major criterion^12–15^. There are other features that should also be taken into account for subclassification, such as the development of two or more different tumors because these cases provide a good model to examine “field effect”. This effect is associated with the tendency of healthy colorectal mucosa to suffer early molecular alterations that trigger malignant transformation of the tissue^16,17^. Synchronous CRC (SCRC) is defined by the presence of more than one tumor simultaneously, while metachronous CRC (MCRC) is characterized by the development of a second lesion after surgery and/or diagnosis of the primary tumor^18–20^.

Given the above, when focusing on CIMP status, it is important to take the type of CRC, into consideration, since CIMP status affects the response to therapy^21,22^ and it may have a relation to the “field effect” linked to CRC^23,24^. For this reason, we examined CIMP status in different CRC subtypes: patients with a single tumor (“unique” CRC), divided into early-onset CRC (EOCRC; age at diagnosis ≤ 45 years old) and patients with late-onset CRC (LOCRC; age at diagnosis > 70 years old); and individuals diagnosed with multiple primary CRC, i.e. patients diagnosed with SCRC or MCRC.

## Methods

### Patients

A total of 229 CRC patients were included in this study at 12 de Octubre Hospital (Madrid, Spain). Among these patients (all of them Caucasian), 59 had EOCRC, 71 had LOCRC, 54 were diagnosed with SCRC and 45 were diagnosed with MCRC. SCRC was diagnosed when two or more histologically different lesions were developed simultaneously or within a time lapse shorter than six months after the detection of the first tumor^19^. When a secondary neoplasm was detected outside the anastomosis area after more than 6 months of the initial tumor diagnosis, it was considered as MCRC^25^. Samples with the highest content of tumor tissue were selected for molecular analysis. A multi-site database was used to collect clinicopathological, therapeutic and pre- and post-operative information. Informed consent was signed by all patients or by a first degree relative when the patient had died. The protocol of this study was approved by the Ethics Committee of this Institution.

### DNA isolation

Formalin-fixed paraffin-embedded (FFPE) tumor tissue samples were selected by a pathologist. To be suitable for molecular analysis, one sample should contain a minimum percentage of 70% tumor cells. Briefly, DNA was extracted from FFPE samples using mineral oil to dissolve the paraffin, followed by proteinase K digestion and ethanol precipitation. DNA was further purified and eluted using CLART^®^ HPV2 kit (Genomica S.A.U., Madrid, Spain).

### Microsatellite analysis and analysis of BRAF mutation, hypermethylation of MLH1 and germline mutations in MMR genes

**Figure 1:**
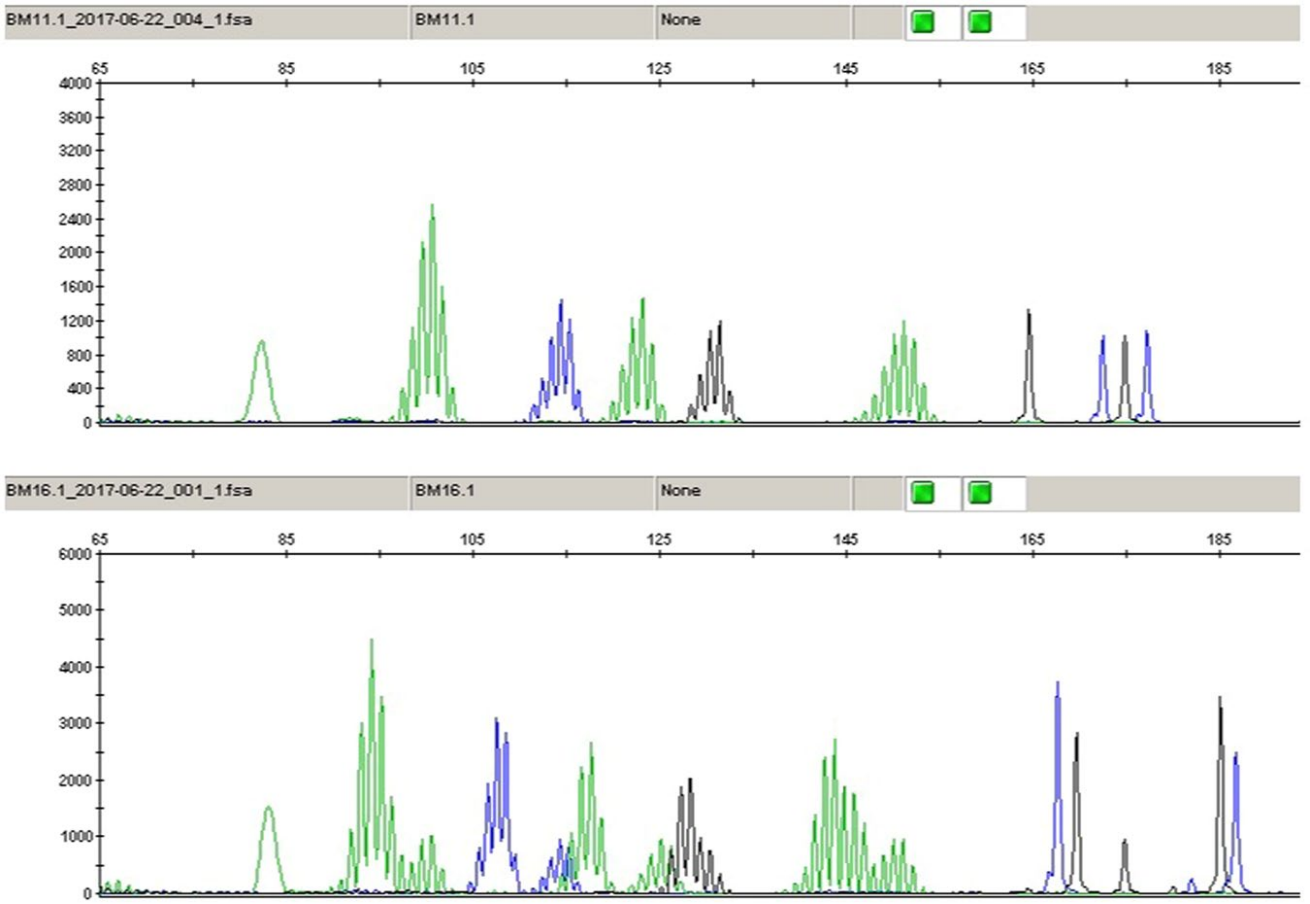
Sample showing Microsatellite stability (above) and other showing Microsatellite instability (below).

Microsatellite instability status of each tumor was defined using the Bethesda five-marker microsatellite panel (NR-21, BAT-26, BAT-25, NR-24 and MONO-27)^26^. Fluorescence labeled primers were included for amplification of markers, then the PCR products were separated using capillary electrophoresis and analyzed. When 2 or more markers showed instability, the sample was defined as microsatellite instable (Fig. 1). Moreover, MSI tumors were analyzed for the *BRAF*^V600E^ mutation and hypermethylation of the *MLH1* gene promoter in order to verify their sporadic nature, using methylation-specific multiplex ligation-dependent probe amplification (MS-MLPA; ME011-B3, ME0042-CIMP, MRC-Holland, Amsterdam, The Netherlands). MSI cases also were screened for Lynch Syndrome by evaluating germline mutations in the MMR genes (*MLH1*, *MSH2* and *MSH6*) by high-resolution melting analysis using a LightCycler 480 real-time PCR system (Roche, Mannheim, Germany), as previously reported^27^.

### KRAS mutations

**Figure 2:**
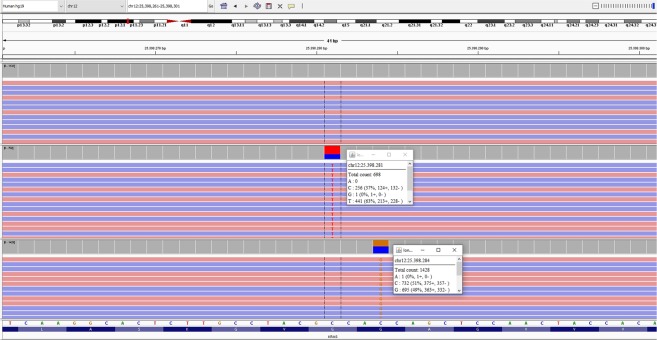
Sample with wild-type *KRAS* (above), other sample with codon 13 mutation (medium), and the last with codon 12 mutation (below), seen in the Integrative Genomics Viewer by Next Generation Sequencing.

Mutations in *KRAS* (codons 12, 13 and 61) were determined previously in patients younger than 45 and older than 70 years. Briefly, DNA was extracted from the neoplastic material, and Sanger sequencing was carried out in both orientations on a 3130 DNA Analyzer (Applied Biosystems, Foster City, CA, USA)^28^. The genetic alterations of *KRAS* in individuals diagnosed with SCRC and MCRC were evaluated by next generation sequencing using a gene panel related to cancer (Ion PGM System, ThermoFisher, Waltham, MA, USA) (Fig. 2).

### CpG island methylator phenotype analysis

**Figure 3:**
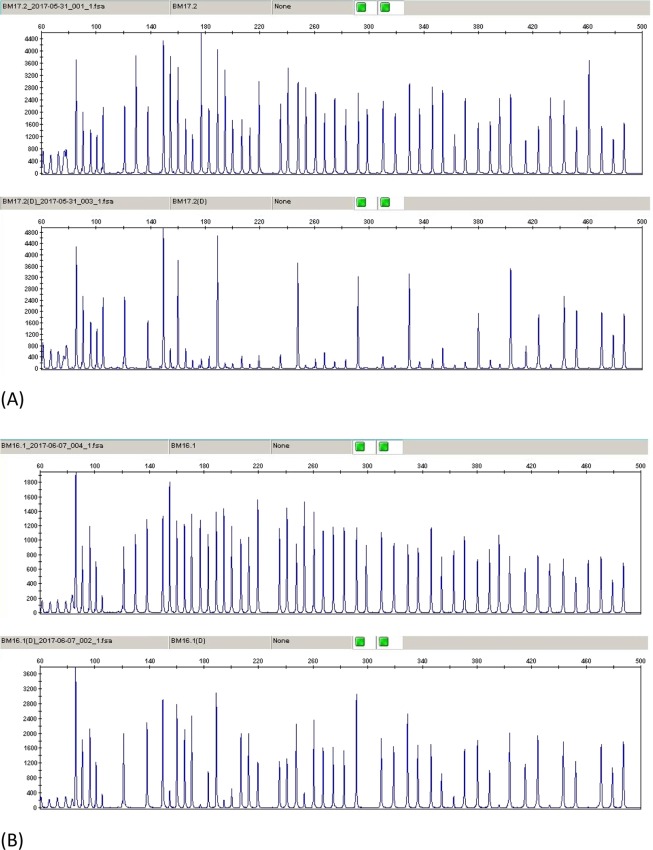
(**A**) Sample with CpG Island Methylator Phenotype negative (CIMP−). (**B**) Sample with CpG Island Methylator Phenotype positive (CIMP+).

For the evaluation of CIMP, we examined the methylation status of the promoter regions of *CACNA1G*, *CDKN2A*, *CRABP1*, *IGF2*, *MLH1*, *NEUROG1*, *RUNX3* and *SOCS1* using the SALSA MS-MLPA Probemix (ME0042-CIMP, MRC-Holland, Amsterdam, The Netherlands). Each patient was classified as CIMP-(+) or CIMP-(−) depending on whether tumors showed ≥ 5/8 or < 5/8 methylated promoters, respectively (Fig. 3A,B)^11^. Patients diagnosed with multiple CRC and distinct CIMP statuses in their tumors were categorized as CIMP-MM (mismatching).

### Statistical analysis

Clinical characteristics were compared between different groups according to CIMP status, including age, sex, stage, tumor location, presence of mucinous features, *BRAF* and *KRAS* mutations, and MSI status. Categorical variables were expressed as number of cases and their percentage, and continuous variables were expressed as mean values plus/minus standard deviation (SD). Comparison of categorical variables was done using Pearson’s Chi Square (X^2^) test. For comparisons between two groups Student’s *t* test was performed, and for comparisons between more than two groups analysis of variance (ANOVA) (for normal distributions) or the Kruskal-Wallis test (for nonparametric distributions) were used. Statistical analysis was carried out using IBM SPSS 17.0 (SPSS Inc., Chicago, IL, USA). P value of <0.05 was considered statistically significant differences.

### Ethics approval and consent to participate

Study approval was obtained from the Ethics Committee of the 12 de Octubre University Hospital in Madrid, Spain. All procedures were performed in accordance with the ethical standards of the institutional research committee and the Declaration of Helsinki.

## Results

### Global features of “unique” colorectal cancer

We collected clinical characteristics of 130 individuals with “unique” CRC, of which 59 were EOCRC cases and the remaining 71 patients were LOCRC cases. The average age at diagnosis was 40 ± 5 and 78 ± 6 years old for EOCRC and LOCRC, respectively. Male to female ratio was >1 in the EOCRC group, whereas in the LOCRC set it was < 1. When we assessed the anatomical location of the tumors, the EOCRC group showed most tumors (47.5%) at the distal colon, while LOCRC patients presented more rectum location (42.3%). Among the 59 patients with EOCRC, 8 (13.6%) patients showed MSI status, of which 3 (37.5%) were sporadic cases: 1 (12.5%) presented a *BRAF* mutation and 2 (25.0%) showed hypermethylation of the *MLH1* gene promoter; the remaining 5 (62.5%) individuals were diagnosed with LS. Moreover, 22 (40.7%) EOCRC patients had a *KRAS* mutation. In the LOCRC group, 6 (8.5%) patients showed MSI status, all of them sporadic cases, 6 (8.5%) patients had a *BRAF* mutation and 1 (16.7%) case presented a *KRAS* mutation (Tables 1 and 2).

**Table 1:** Clinical variables of interest and CIMP status in the EOCRC.

	Total	Tumor CIMP-(−)	Tumor CIMP-(+)	p-value^a^
No. of patients	59 (100.0)	50 (84.7)	9 (15.2)	—
Average age of onset	40 [5]	40 [5]	39 [5]	NS^b^
**Sex**				
Female	23 (39.0)	19 (62.0)	4 (44.4)	NS
Male	36 (61.0)	31 (38.0)	5 (55.6)	
**Location**				
Proximal colon	11 (18.6)	6 (12.0)	5 (55.6)	0.008
Distal colon	28 (47.5)	26 (52.0)	2 (22.2)	
Rectum	20 (33.9)	18 (36.0)	2 (22.2)	
**Tumor histology**				
Adenocarcinoma	47 (79.7)	40 (80.0)	7 (77.8)	NS
Adenoma with HGD	12 (20.3)	10 (20.0)	2 (22.2)	
**Tumor stage**				
I	16 (27.2)	13 (26.0)	3 (33.3)	NS
II	23 (39.0)	19 (38.0)	4 (44.4)	
III	10 (16.9)	9 (18.0)	1 (11.1)	
IV	10 (16.9)	9 (18.0)	1 (11.1)	
**Tumor differentiation**				
Well differentiated	18 (38.3)	17 (41.5)	1 (16.7)	NS
Moderately differentiated	24 (51.1)	21 (51.2)	3 (50.0)	
Poorly differentiated	5 (10.6)	3 (7.3)	2 (33.3)	
Mucin production Tumor	15 (31.9)	12 (29.3)	3 (50.0)	NS
“Signet ring” cells Tumor	3 (7.3)	3 (7.3)	—	NS
**Microsatellite status**				
MSS	51 (86.4)	43 (86.0)	8 (88.9)	NS
MSI	8 (13.6)	7 (14.0)	1 (11.1)	
***BRAF***				
Wild type	58 (98.3)	49 (98.0)	9 (100.0)	NS
Mutated	1 (1.7)	1 (2.0)	—	
***KRAS***				
Wild type	32 (59.3)	27 (58.7)	5 (62.5)	NS
Mutated	22 (40.7)	19 (41.3)	3 (37.5)	
**Familial cancer history**				
Sporadic	31 (52.5)	26 (52.0)	5 (55.6)	NS
Familial aggregation	23 (39.0)	20 (40.0)	3 (33.3)	
HNPCC	5 (8.5)	4 (8.0)	1 (11.1)	

^a^Statistical comparison was performed using Pearson’s Chi Square test (χ^2^). ^b^Statistical comparison was performed using Student’s *t-*test. Parenthesis refer to percentage numbers. Brackets are used to identify standard deviation. Percentages come from different initial number of patients because some cases were excluded: when only one biopsy was available or in cases of carcinoma *in situ* with severe dysplasia in which other features could not be studied. CIMP: CpG island methylator phenotype. No. Number. HGD: High-grade dysplasia. MSI: Microsatellite instability. MSS: Microsatellite stability. HNPCC: Hereditary non-polyposis colorectal cancer. NS: Not significant.

**Table 2:** Clinical variables of interest and CIMP status in the LOCRC subgroup.

	Total	Tumor CIMP-(−)	Tumor CIMP-(+)	p-value^a^
No. of patients	71 (100.0)	52 (73.2)	19 (26.8)	NS^b^
Average age of onset	78 [6]	78 [6]	79 [5]	NS
**Sex**				
Female	38 (53.5)	26 (50.0)	12 (63.2)	NS
Male	33 (46.5)	26 (50.0)	7 (36.8)	
**Location**				
Proximal colon	28 (39.4)	22 (42.3)	6 (31.6)	NS
Distal colon	13 (18.3)	12 (23.1)	1 (5.3)	
Rectum	30 (42.3)	18 (34.6)	12 (63.2)	
**Tumor histology**				
Adenocarcinoma	71 (100.0)	52 (100.0)	19 (100.0)	NS
Adenoma with HGD	—	—	—	
**Tumor stage**				
I	3 (4.3)	3 (6.0)	—	NS
II	34 (49.3)	24 (48.0)	10 (52.6)	
III	18 (26.1)	15 (30.0)	3 (15.8)	
IV	14 (20.3)	8 (16.0)	6 (31.6)	
**Tumor differentiation**				
Well differentiated	16 (24.2)	15 (30.6)	1 (5.9)	0.045
Moderately differentiated	47 (71.2)	33 (67.4)	14 (82.4)	
Poorly differentiated	3 (4.6)	1 (2.0)	2 (11.8)	
Mucin production Tumor	11 (16.7)	9 (18.4)	2 (11.8)	NS
“Signet ring” cells Tumor	2 (3.0)	2 (4.1)	—	NS
**Microsatellite status**				
MSS	65 (91.5)	50 (96.2)	15 (78.9)	0.021
MSI	6 (8.5)	2 (3.8)	4 (21.1)	
***BRAF***				
Wild type	65 (91.5)	51 (98.1)	14 (73.7)	0.001
Mutated	6 (8.5)	1 (1.9)	5 (26.3)	
***KRAS***				
Wild type	5 (83.3)	4 (80.0)	1 (100.0)	NS
Mutated	1 (16.7)	1 (20.0)	—	
**Familial cancer history**				
Sporadic	62 (91.2)	45 (90.0)	17 (94.4)	NS
Familial aggregation	6 (8.8)	5 (10.0)	1 (5.6)	
HNPCC	3 (4.4)	2 (4)	1 (5.6)	

^a^Statistical comparison was performed using Pearson’s Chi Square test (χ^2^). ^b^Statistical comparison was performed using Student’s *t-*test. Parenthesis refer to percentage numbers. Brackets are used to identify standard deviation. Percentages come from different initial number of patients because some cases were excluded: when only one biopsy was available or in cases of carcinoma *in situ* with severe dysplasia in which other features could not be studied. CIMP: CpG island methylator phenotype. No.: Number. HGD: High-grade dysplasia. MSI: Microsatellite instability. MSS: Microsatellite stability. HNPCC: Hereditary non-polyposis colorectal cancer. NS: Not significant.

### Global features of primary multiple colorectal cancer

Among 99 cases with multiple CRC, 54 individuals were diagnosed with SCRC and 45 patients with MCRC. In both groups, the mean age at onset was around 70 years old, with a male to female ratio > 1. Moreover, the most common tumor location for both groups was the entire colon, defined as the location of the synchronous or metachronous tumors at different sides of the colon. Only one patient in the SRCR group (1.8%) had MSI in both tumors whereas discordant MSI status was found between synchronous tumors in 3 cases (5.6%), of which 1 (25.0%) case was diagnosed with LS. Five (10.4%) and 31 (64.6%) patients presented *BRAF* mutations and *KRAS* mutations in at least one tumor, respectively. In the MCRC group, several remarkable features were the diagnosis of metachronous neoplasm at an early stage (82.2%), the total concordance of MSI status between paired tumors, and only 1 (2.2%) patient who was identified as a LS case, showed MSI status. Regarding a *BRAF* mutation, MCRC patients showed concordance between both tumors, of which 1 (2.2%) patient presented a *BRAF* mutation. Eighteen (40.0%) MCRC patients showed a *KRAS* mutation in at least one tumor and 11 (24.4%) in paired-tumors (Tables 3 and 4).

**Table 3:** Clinical variables of interest and CIMP status in the SCRC subgroup.

	Total	Tumor A CIMP-(−)	Tumor A CIMP-(+)	Tumor A	p-value^a^
		Tumor B CIMP-(−)	Tumor B CIMP-(+)	Tumor B CIMP-(MM)	
No. of patients	54 (100.0)	19 (35.2)	20 (37.0)	15 (27.8)	—
Average age of onset	70 [10]	72 [10]	67 [11]	71 [10]	NS^b^
**Sex**					
Female	18 (33.3)	9 (47.4)	4 (20.0)	5 (33.3)	NS
Male	36 (66.7)	10 (52.6)	16 (80.0)	10 (66.7)	
**Location**					
Proximal colon	7 (13.0)	3 (15.8)	1 (5.0)	3 (20.0)	NS
Distal colon	21 (38.9)	6 (31.6)	11 (55.0)	4 (26.7)	
Entire colon	26 (48.1)	10 (52.6)	8 (40.0)	8 (53.3)	
**Tumor histology**					
Adenocarcinoma	44 (81.5)	14 (73.7)	17 (85.0)	13 (86.7)	NS
Adenoma with HGD	10 (18.5)	5 (26.3)	3 (15.0)	2 (13.3)	
**Tumor stage**					
I	24 (44.4)	9 (47.4)	7 (35.0)	8 (53.3)	NS
II	17 (31.5)	8 (42.1)	5 (25.0)	4 (26.7)	
III	10 (18.5)	2 (10.5)	6 (30.0)	2 13.3)	
IV	3 (5.6)	—	2 (10.0)	1 (6.7)	
**Tumor differentiation**					
Well differentiated	23 (52.3)	10 (71.4)	7 (41.2)	6 (46.2)	NS
Moderately differentiated	18 (40.9)	4 (28.6)	9 (52.9)	5 (38.5)	
Poorly differentiated	3 (6.8)	—	1 (5.9)	2 (15.4)	
Mucin production Tumor	10 (23.3)	4 (28.6)	2 (12.5)	4 (30.8)	NS
“Signet ring” cells Tumor	2 (4.6)	1 (7.1)	—	1 (7.7)	NS
**Microsatellite status**					
MSS	50 (92.6)	17 (89.5)	20 (100.0)	13 (86.7)	NS
MSI	1 (1.8)	1 (5.3)	—	—	
MSS & MSI	3 (5.6)	1 (5.3)	—	2 (13.3)	
***BRAF***					
Wild type	43 (89.6)	14 (87.5)	17 (94.4)	12 (58.7)	NS
Mutated	1 (2.1)	—	—	1 (7.1)	
Wild type & Mutated	4 (8.3)	2 (12.5)	1 (5.6)	1 (7.1)	
***KRAS***					
Wild type	17 (35.4)	4 (25.0)	7 (39.8)	6 (42.9)	NS
Mutated	12 (25.0)	6 (37.5)	3 (16.7)	3 (21.4)	
Wild type & Mutated	19 (39.6)	6 (37.5)	8 (44.4)	5 (35.7)	
**Familial Cancer History**					
Sporadic	44 (81.5)	15 (78.9)	16 (80.0)	13 (86.6)	NS
Familial aggregation	9 (16.7)	4 (21.1)	4 (20.0)	1 (6.7)	
HNPCC	1 (1.8)	—	—	1 (6.7)	

^a^Statistical comparison was performed using Pearson’s Chi Square test (χ^2^). ^b^Statistical comparison was performed using analysis of variance (ANOVA). Parenthesis refer to percentage numbers. Brackets are used to identify standard deviation. Percentages come from different initial number of patients because some cases were excluded: when only one biopsy was available or in cases of carcinoma *in situ* with severe dysplasia in which other features could not be studied. CIMP: CpG island methylator phenotype. No.: Number. HGD: High-grade dysplasia. MSI: Microsatellite instability. MSS: Microsatellite stability. HNPCC: Hereditary non-polyposis colorectal cancer. NS: Not significant.

**Table 4:** Clinical variables of interest and CIMP status in the MCRC subgroup.

	Total	1^st^ Tumor CIMP-(−) 2^nd^ Tumor CIMP-(−)	1^st^ Tumor CIMP-(−) 2^nd^ Tumor CIMP-(+)	1^st^ Tumor CIMP-(+) 2^nd^ Tumor CIMP-(−)	1^st^ Tumor CIMP-(+) 2^nd^ Tumor CIMP-(+)	p-value^a^
No. of patients	45 (100.0)	16 (35.6)	9 (20.0)	9 (20.0)	11 (24.4)	—
**Average age of onset**						
1^st^ Tumor	69 [8]	71 [8]	68 [12]	66 [8]	66 [5]	NS^b^
2^nd^ Tumor	72 [8]	74 [7.9]	71 [12.5]	70 [7]	70 [5.5]	NS^b^
**Sex**						
Female	16 (35.6)	5 (31.2)	6 (66.7)	3 (33.3)	2 (18.2)	NS
Male	29 (64.4)	11 (68.8)	3 (33.3)	6 (66.7)	9 (81.8)	
**Location**						
Proximal colon	5 (11.1)	2 (12.5)	1 (11.1)	1 (11.1)	1 (9.1)	NS
Distal colon	10 (22.2)	6 (37.5)	2 (22.2)	1 (11.1)	1 (9.1)	
Entire colon	30 (66.7)	8 (50.0)	6 (66.7)	7 (77.8)	9 (81.8)	
**1** ^**st**^ **Tumor histology**						
Adenocarcinoma	28 (62.2)	10 (62.5)	4 (44.4)	8 (88.9)	6 (54.5)	NS
Adenoma with HGD	17 (37.8)	6 (37.5)	5 (55.6)	1 (11.1)	5 (45.5)	
**1** ^**st**^ **Tumor stage**						
0	16 (35.6)	6 (37.5)	5 (55.6)	1 (11.1)	4 (36.4)	NS
I	3 (6.7)	1 (6.3)	—	—	2 (18.2)	
II	13 (28.8)	3 (18.8)	3 (33.3)	6 (66.7)	1 (9.1)	
III	10 (22.2)	4 (25.0)	1 (11.1)	1 (11.1)	4 (36.4)	
IV	3 (6.7)	2 (12.5)	—	1 (11.1)	—	
**1** ^**st**^ **Tumor differentiation**						
Well differentiated	30 (83.3)	11 (91.7)	6 (85.7)	6 (66.7)	7 (87.5)	NS
Moderately differentiated	6 (16.7)	1 (8.3)	1 (14.3)	3 (33.3)	1 (12.5)	
Poorly differentiated	—	—	—	—	—	
Mucin production 1^st^ Tumor	3 (10.3)	1 (8.3)	—	1 (11.1)	2 (25.0)	NS
“Signet ring” cells 1^st^ Tumor	1 (3.8)	—	—	1 (11.1)	—	NS
**2** ^**nd**^ **Tumor histology**						
Adenocarcinoma	8 (17.8)	—	2 (22.2)	2 (22.2)	4 (36.4)	NS
Adenoma with HGD	37 (82.2)	16 (100)	7 (77.8)	7 (77.8)	7 (63.6)	
**2** ^**nd**^ **Tumor stage**						
0	35 (77.8)	14 (87.5)	7 (77.8)	7 (77.8)	7 (63.6)	NS
I	2 (4.4)	1 (6.3)	—	—	1 (9.1)	
II	7 (15.6)	1 (6.3)	2 (22.2)	2 (22.2)	2 (18.2)	
III	1 (2.2)	—	—	—	1 (9.1)	
IV	—	—	—	—	—	
**2** ^**nd**^ **Tumor differentiation**						
Well differentiated	14 (77.8)	5 (100.0)	3 (75.0)	1 (50.0)	5 (71.4)	NS
Moderately differentiated	3 (16.6)	—	—	1 (50.0)	2 (28.6)	
Poorly differentiated	1 (5.6)	—	1 (25.0)	—	—	
Mucin production 2^nd^ Tumor	2 (13.3)	—	—	1 (25.0)	1 (14.3)	NS
“Signet ring” cells 2^nd^ Tumor	—	—	—	—	—	—
**Microsatellite status**						
MSS	44 (97.8)	16 (100)	9 (100)	9 (100)	10 (90.9)	NS
MSI	1 (2.2)	—	—	—	1 (9.1)	
***BRAF***						
Wild type	44 (97.8)	16 (100)	8 (88.9)	9 (100)	11 (100)	NS
Mutated	1 (2.2)	—	1 (11.1)	—	—	
***KRAS***						
Wild type	16 (35.6)	7 (43.8)	3 (33.3)	4 (44.4)	2 (18.2)	NS
Mutated	11 (24.4)	1 (6.2)	3 (33.3)	3 (33.3)	4 (36.4)	
Wild type & Mutated	18 (40.0)	8 (50.0)	3 (33.3)	2 (22.2)	5 (45.5)	
**Familial Cancer history**						
Sporadic	35 (77.8)	12 (75.0)	5 (55.6)	8 (88.9)	10 (90.9)	NS
Familial aggregation	9 (20.0)	4 (25.0)	4 (44.4)	1 (11.1)	—	
HNPCC	1 (2.2)	—	—	—	1 (9.1)	

^a^Statistical comparison was performed using Pearson’s Chi Square test (χ^2^). ^b^Statistical comparison was performed using analysis of variance (ANOVA). Parenthesis refer to percentage numbers. Brackets are used to identify standard deviation. Percentages come from different initial number of patients because some cases were excluded: when only one biopsy was available or in cases of carcinoma *in situ* with severe dysplasia in which other features could not be studied. CIMP: CpG island methylator phenotype. No.: Number. HGD: High-grade dysplasia. MSI: Microsatellite instability. MSS: Microsatellite stability. HNPCC: Hereditary non-polyposis colorectal cancer. NS: Not significant.

### CIMP analysis

**Figure 4:**
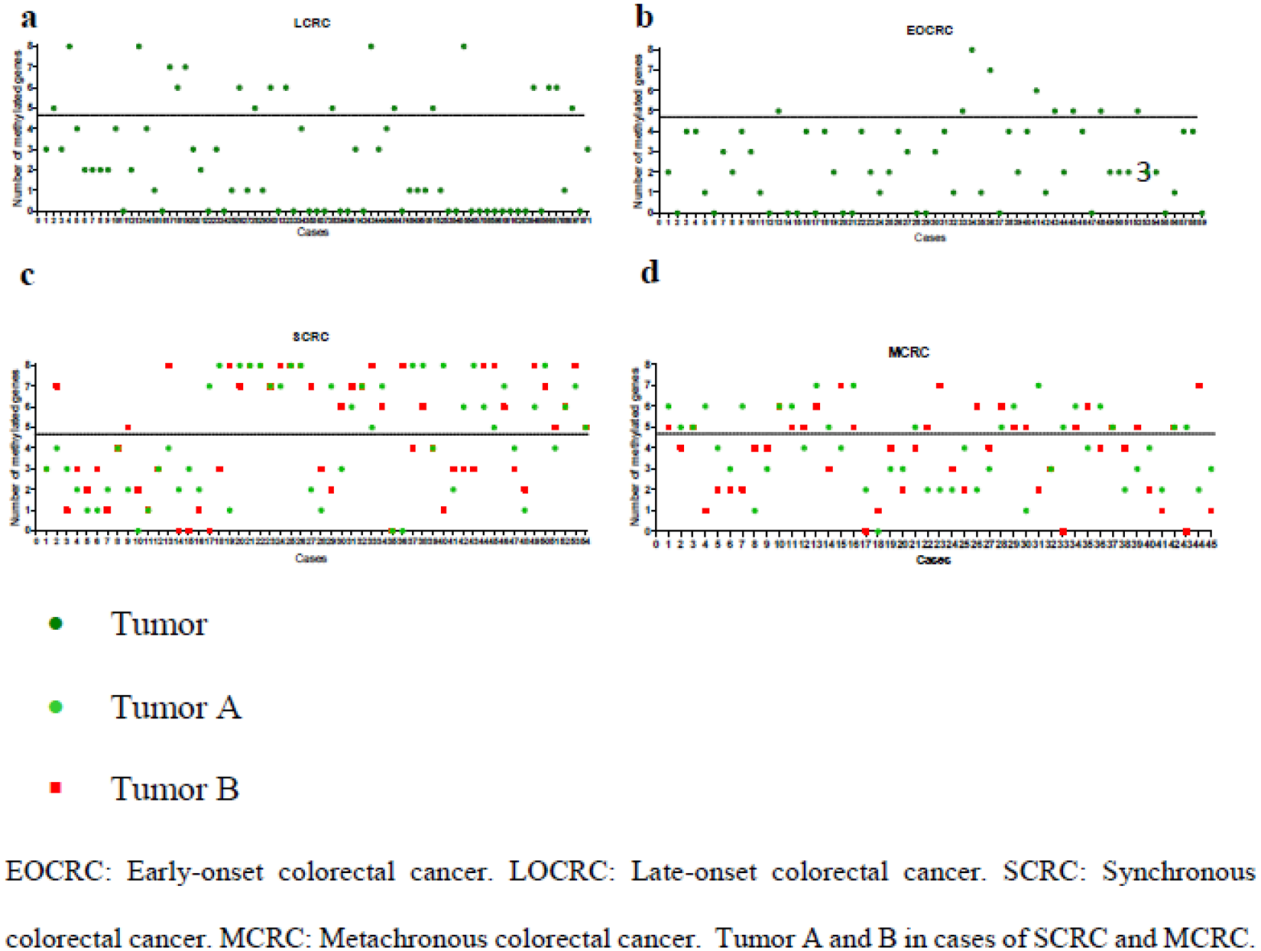
Number of methylated genes in each tumor of (**a**) EOCRC patients; (**b**) LOCRC patients; (**c**) SCRC patients; (**d**) MCRC patients.

We analyzed CIMP status in the four subtypes of CRCs patients. The number of methylated genes in each tumor of the patients of the different subgroups is shown in Fig. 4. In the cohort of 59 patients with EOCRC, 50 (84.7%) tumors were CIMP-(−) and 9 (15.2%) were CIMP-(+). In the 71 patients with LOCRC, 52 (73.2%) tumors were CIMP-(−) and 19 (26.8%) tumors were CIMP-(+) (Tables 1 and 2). Interestingly, the subset of 54 diagnosed individuals with SCRC showed 20 (37.0%) patients with CIMP-(+) tumors and 19 (35.2%) with CIMP-(−) tumors, where both tumors presented same CIMP status, and 15 (27.8%) patients with CIMP-MM tumors. In the cohort of 45 patients with MCRC, 11 (24.4%) were CIMP-(+) for both tumors, 16 (35.6%) were CIMP-(−) for paired tumors, and 18 (40.0%) showed CIMP-MM: 9 (20.0%) were first tumor CIMP-(+) and second tumor CIMP-(−), and 9 (20.0%) were first tumor CIMP-(−) and second tumor CIMP-(+) (Tables 3 and 4). Furthermore, the concordance of CIMP status between paired tumors in the SCRC and MCRC groups is summarized in Tables 5 and 6. Thirty-nine (72.2%) SCRC patients showed the same CIMP status in both tumors. In the cohort of MCRC, 27 (60%) patients presented concordant CIMP status in both tumors.

**Table 5:** Concordance of CIMP status in paired-tumors from patients diagnosed with SCRC using eight-marker panel.

CIMP status	Tumor B (+)	Tumor B (−)
Tumor A (+)	20 (37.0%)	1 (1.9%)
Tumor A (−)	14 (25.9%)	19 (35.2%)

CIMP: CpG island methylator phenotype. SCRCs: Synchronous colorectal cancer.

**Table 6:** Concordance of CIMP status in paired-tumors from patients diagnosed with MCRC using eight-marker panel.

CIMP status	2^nd^ Tumor (+)	2^nd^ Tumor (−)
1^st^ Tumor (+)	11 (24.4%)	9 (20.0%)
1^st^ Tumor (−)	9 (20.0%)	16 (35.6%)

CIMP: CpG island methylator phenotype. MCRCs: Metachronous colorectal cancers.

### Correlation between CIMP phenotype and clinical features

Regarding clinical correlation with CIMP status, CIMP-(+) tumors in the EOCRC group were mainly located in proximal colon (p = 0.008), with a very low rate of well-differentiated tumors (Table 1). The CIMP-(+) tumors of patients with LOCRC presented moderate tumor differentiation (p = 0.045), MSI status (p = 0.021) and a higher *BRAF* mutation rate (p = 0.001) (Table 2). Other interesting features were the higher proportion of females as well as the rectal tumor location (both 63%). In the SCRC subgroup (Table 3), cases with both tumors CIMP-(+), 16 (80.0%) patients were male and 11 (55.0%) tumors were distal-sided both paired tumors. Most patients diagnosed with MCRC and CIMP-(+) for both tumors showed entire colon location (81.8%), mucin production in the first tumor (25%) and early stage at diagnosis of the second tumor (72.7%) (Table 4). Differences in diagnostic tumor stage, sex, age and other clinicopathological features analyzed between different and concordant CIMP status in the SCRC and MCRC groups were not statistically significant (Tables 3 and 4).

## Discussion

CpG-island methylation testing has been proposed as a tool for cancer detection and prognosis, and indicates that methylation status is of clinical relevance^29^, despite the timing of its occurrence and its interaction with other genetic defects are not fully understood^30–32^. In this study, we assessed CIMP status in different subsets of CRCs according to age of onset and the number of primary neoplasms: EOCRC and LOCRC with “unique” CRCs, and SCRC and MCRC.

In the assessment of CIMP status^11,33^, we found that only 15.2% of EOCRC patients and 26.8% of LOCRC patients showed CIMP-(+) tumors. However, 37.0% of SCRC patients were CIMP-(+) for both tumors and 27.8% of the tumor pairs showed at least one CIMP-(+) tumor, suggesting that the serrated pathway of carcinogenesis could be the main mechanism for SCRC development^34,35^. In the MCRC group, 24.4% were CIMP-(+) for both tumors and 40.0% were CIMP-(+) for at least one tumor. The reported differences in CIMP-(+) frequency between different groups could reflect the relative involvement of this particular molecular phenotype throughout the development of multiple neoplasms. In summary, our findings show a higher CIMP-(+) frequency in patients who develop multiple tumors than in patients who develop a single tumor. These different epigenetic patterns may be due to a “field effect” and possibly a highly-susceptible tissue microenvironment could be generated by the interplay of different etiologic factors including lifestyle, eating habits or environmental issues leading to the appearance and development of malignant tumors^20,36^.

Moreover, in this study we observed that most pairs of tumors had concordant CIMP status in patients with SCRC (72.2%) which suggests that CIMP is maintained throughout neoplasm development; this has been taken as a evidence for the high probability that synchronous tumors could be developed through the same genetic pathway in each particular patient^37^. On the other hand, 40% of the patients diagnosed with MCRC did not present concordance concerning CIMP status. The clinical implication of this finding is that the analysis of the CIMP status in any of the synchronous tumors could provide a reasonably reliable prediction of CIMP status in the other tumors even when it is not directly assayed. However, this prediction would be less reliable in MCRC. Comparison of these epigenetic patterns in synchronous and metachronous lesions may suggest that there is a tendency to have clonal features or a stronger “field effect” in patients diagnosed with SCRC, while the tumor heterogeneity in patients with MCRC may be caused by the contribution of different carcinogenic pathways in tumors developed at different time points.

About the results from the correlation between CIMP status and the clinical features within each group of tumors, recent systematic reviews have confirmed the association between CIMP phenotype and older ages, female gender, proximal tumor location, mucinous histology, poor differentiation and MSI^36,37^. According to the “unique” colorectal cancers, EOCRC subset showed the proximal location, mucinous and poorly-differentiated phenotype, while LOCRC linked with a higher proportion within this subset, and gender and MSI phenotype. Maybe the age-of-onset criterion gives rise to this stratification in the characteristics related to the CIMP in this type of CRC. Nevertheless, this characteristics didn´t correlate with Multiple Primary CRCs.

Our results seem to confirm the fact that there are distinct groups of CRC patients. This study, focused on the current knowledge of epigenetic alterations in CRC, could represent a substantial contribution to this research line, since it may have implications in terms of prevention, diagnosis and therapy.

## Conclusion

Our results underscore the importance of taking into account several criteria for the development of multiple primary tumors when analyzing CRC. There is higher CIMP-(+) frequency in patients diagnosed with multiple CRC than in patients with “unique” CRC. Additionally, we conclude that there is a concordance of CIMP status of synchronous tumors in SCRC. Therefore, it could be suggested that CIMP status in one of the simultaneous tumors could predict CIMP status of other tumors.

## Data Availability

The data that support the findings of this study are available from the corresponding author on reasonable request.
